# Formation mechanisms and environmental influences on the crystal growth of wulfenite

**DOI:** 10.1038/s41598-024-60043-4

**Published:** 2024-04-22

**Authors:** Nik Gračanin, Matejka Podlogar, Sorour Semsari Parapari, Pascal Boulet, Francisco Ruiz-Zepeda, Sašo Šturm, Nastja Rogan Šmuc

**Affiliations:** 1 Department for Nanostructured Materials, Jozef Stefan Institute, Jamova Cesta 39, 1000 Ljubljana, Slovenia; 2 Department of Geology, Faculty of Natural Sciences and Engineering, University of Ljubljana, Aškerčeva 12, 1000 Ljubljana, Slovenia; 3 Institut Jean Lamour (UMR CNRS 7198), Centre de Compétences XGamma, 2, Allée André Guinier, 54011 Nancy, France; 4 Department of Materials Chemistry, National Institute of Chemistry, Hajdrihova 19, 1000 Ljubljana, Slovenia

**Keywords:** Environmental sciences, Solid Earth sciences, Materials science

## Abstract

In this study, we introduce a novel approach using correlative analysis techniques to unravel detailed insights into the environmental influences on crystal growth. Tabular and bipyramidal wulfenite samples from the Mežica mine in north-eastern Slovenia were analysed to combine the morphological aspects of crystal growth with the atomic-resolution reconstruction of the positions of lead (Pb) and molybdenum (Mo) atoms in the parent crystal lattice. These combined data also allow us to present the formation mechanism that enables the development of bipyramidal or tabular morphologies in wulfenite. The bipyramidal and tabular crystals are chemically pure wulfenite (PbMoO_4_), as confirmed by various advanced diffraction and spectroscopy techniques. However, each habit includes multiple inclusions, mostly consisting of carbonates, Pb-Fe oxides, Pb oxides and, more rarely, Pb vanadate (descloizite). The differences in the morphologies can be attributed to compositional changes during precipitation from a meteoric solution and thus, we propose a growth mechanism consisting of three different phases of growth. This innovative approach emphasises the importance of understanding the origin of crystal habits, as can help to decipher how external influences can affect the crystal structure and its surface, leading to the dissolution of preferred surfaces and the selective release of Pb and Mo.

## Introduction

In this scientific research, we introduce wulfenite crystals with a wide range of crystal habits from the Mežica area (Republic of Slovenia). Wulfenite crystals provide an excellent opportunity to study and understand the relationship between their crystal habits and the underlying crystal chemistry, structure, and geological formation environment.

The mineral wulfenite (PbMoO_4_) is a member of the scheelite-type group of molybdates and tungstates, with the general formula of ABO_4_, where A is a divalent metal cation with an ionic radius > 0.90 Å and B is a highly charged cation (W^6+^ or Mo^6+^). The scheelite subgroup with Pb as a cation is a solid solution series, with wulfenite (PbMoO_4_) and stolzite (PbWO_4_) being the end members; this subgroup has no known miscibility gap at ambient conditions and Mo^6+^ can be freely substituted by W^6+^ and other elements, such as As, V, or Cu^1,2^. This mineral group has tetragonal symmetry and belongs to space group I4_1_/a, with four formula units per unit cell (Z = 4). The unit cell of wulfenite comprises (MoO_4_)^2−^ anions, where each Mo is surrounded by four O atoms forming a tetrahedron. Pb^2+^ cations are connected to eight adjacent MoO_4_ tetrahedra via Pb–O bonds and forms an eight coordinated PbO_8_ unit^3^. PbO_8_ unit can be described as two concentric tetrahedra, one flattened along [001] and the second one rotated relative to the first one and elongated in the [001] direction^4^. The eight corner O atoms of PbO_8_ are linked to eight MoO_4_ tetrahedra^3^. Curiously, wulfenite crystals exhibit a very diverse external morphology, with bipyramidal (with {101} as the most distinct crystallographic surfaces) and tabular crystals (with {001} as the most distinct surfaces) being the most common^5^. Mežica wulfenites can have bipyramidal, thin tabular to thick tabular, and, most interestingly, hemimorphic pyramidal crystals, which is very unusual for minerals crystallising in space group I4_1_/a [n°88] but common in space groups with lower symmetry, such as I4̅ [n°82]^6–8^. Lower symmetry was proposed to occur in the series of stolzite-wulfenite solid solutions^5,9,10^, but so far, the minerals in this series have been confirmed to retain space group I4_1_/a^11^. The different morphologies could be due to growth factors, i.e., the growth rates of the crystals depend on the change in the chemical composition of the solutions from which the wulfenite crystals precipitate and the spatial orientation of the crystals during their growth^12^.

Vesselinov^9,10,12^ studied in detail the growth-related morphological diversity of wulfenite crystals by analysing both natural wulfenite samples from the Mežica Mine and laboratory-grown wulfenite crystals. Vesselinov discovered that the morphology of laboratory-grown wulfenite crystals precipitated from an aqueous solution at ambient temperature and atmospheric pressure is influenced by the Pb/Mo concentration ratio. In particular, higher Pb concentrations promote bipyramidal shapes, while higher MoO_2_^−4^ values favour tabular crystals^12^.

Wulfenite crystals (both bipyramidal and tabular) from Mežica contain primary fluid inclusions formed during the crystallisation of the wulfenite. The inclusions are single-phase, low-mineralised water, as determined by Raman spectroscopy^11^. The presence of these inclusions suggests that the wulfenite from Mežica was precipitated from low-temperature (< 50 °C) mineralised brines (aqueous NaCl-CaCl_2_ solution) of meteoric origin^11,13^. These results confirm that the precipitation of the wulfenite crystals from Mežica occurred at ambient temperature and atmospheric pressure, which is consistent with the conditions of the wulfenites grown by Vesselinov in the laboratory.

The presented study aims to define well-linked analytical information across multiple length-scale orders, from the millimetre-scale morphological aspects of crystal growth to the micrometre-scale chemical composition of the crystal, leading to an atomically resolved reconstruction of the positions of Pb and Mo atoms in the lattice of the parent crystal. We hypothesised that the combined data would allow us to formulate the mechanism leading to the development of bipyramidal or tabular crystal habits for wulfenite, using Vesselinov’s findings as a theoretical basis.

This innovative approach and comprehensive methodology highlights the importance of understanding the origin of crystal habits and their relationship with environment. Furthermore, this knowledge can help to decipher how external influences can affect the crystal structure and its surface, leading to the dissolution/weathering of preferential surfaces and consequently to the selective release of Pb and Mo. This knowledge is therefore also particularly valuable for environmental scientists and various technical fields.

## Materials and methods

### Study area

The Mežica Mine is a Pb–Zn ore deposit located in north-western Slovenia, in the eastern part of the Karavanke Mountains, which belong to the Eastern Alps. The deposit is situated in Mesozoic sedimentary rocks, which mainly consist of carbonates. The minerals galena (Pb ore) and sphalerite (Zn ore) precipitated from hydrothermal solutions, making Mežica an epigenetic hydrothermal ore deposit that was later diagenetically regenerated^5^. The mineral wulfenite is common throughout the deposit. It is a secondary mineral formed after the oxidation of the primary galena and sphalerite ore deposits in the presence of oxygenated meteoric water^5^.

### Crystal characteristics

**Figure 1:**
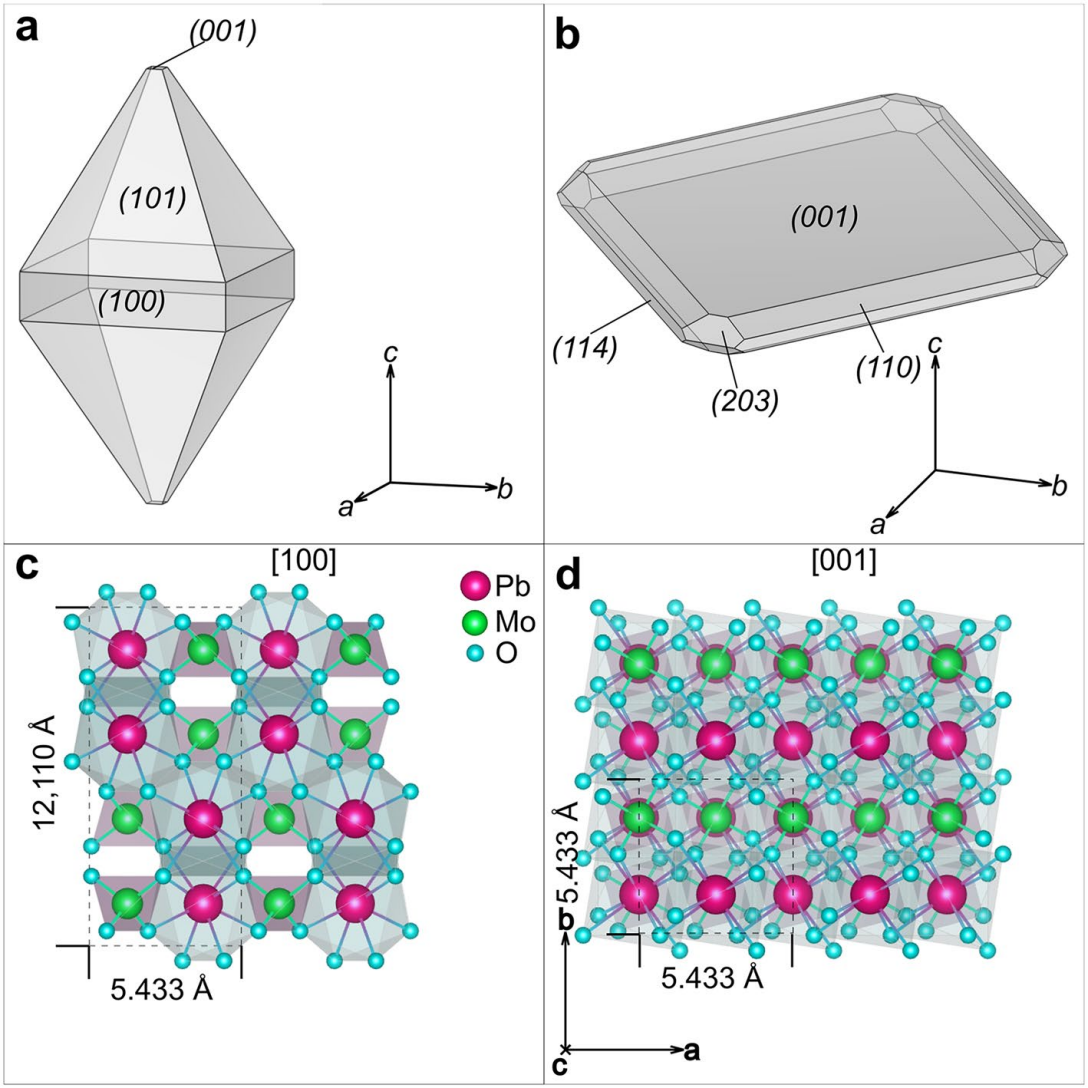
Schematic representation of the two most common morphological varieties of wulfenite crystals, (**a**) bipyramidal and (**b**) tabular crystals, with indexed surfaces. Ideal wulfenite crystal structure viewed along the (**c**) [100] and (**d**) [001] zone axes, respectively.

The selected specimens from the Mežica Mine, which exhibit well-defined bipyramidal and tabular crystal morphologies, were selected for comprehensive chemical and structural analysis. Each specimen, sourced from a druse of wulfenite crystals, is depicted in Fig. 1. Although at least one crystallographic surface was damaged or not fully developed in these specimens, the {110} (bipyramidal) and {001} (tabular) surfaces were still distinctly identifiable. The bipyramidal specimen, extending up to 5 mm along its longest axis (c-axis), had a dark orange tabular band visible on the {100} plane. Moreover, it displayed well-defined and undamaged {010} and {101} surfaces. On the other hand, the tabular crystals, less than 5 mm in thickness, possessed a well-developed {001} surface.

### Analytical methods and specimen preparation

Single-crystal X-ray diffraction (SCXRD) (Institute Jean Lamour, X-Gamma competence center, Nancy, France) was performed using a Kappa APEX II diffractometer (Bruker SAINT is included in the APEX Suite software^14^, Bruker AXS Inc., Madison, Wisconsin, USA, URL link: https://www.bruker.com/en/products-and-solutions/diffractometers-and-x-ray-microscopes/single-crystal-x-ray-diffractometers/sc-xrd-software/apex.html) using MoKα1 monochromatic radiation. Frame integration was executed using the Bruker SAINT software by employing a narrow-frame algorithm. The bipyramidal specimen, an orange translucent crystal measuring approximately 0.020 mm × 0.020 mm × 0.030 mm, was analysed over a span of 16.68 h. The maximum angle (θ) and the resolution were set to 55.55° and 0.43 Å, respectively, resulting in 15,252 reflections. Meanwhile, the tabular specimen, an orange-yellowish translucent crystal with dimensions of roughly 0.050 mm × 0.050 mm × 0.060 mm, was examined over 18.99 h, achieving a maximum θ angle of 68.87° and a resolution of 0.38 Å. This resulted in 14,208 reflections. Data refinement was performed using a full-matrix least squares method on F^2^, with the SHELXL-2014/7 software (SHELXL Version 2014/7, Crystal structure refinement with SHELXL^15^, URL link: http://shelx.uni-goettingen.de).

Detailed morphological, structural, and semi-quantitative chemical analyses of wulfenite samples were performed using a Thermo Fisher Quanta 650 tungsten-emission-gun environmental scanning electron microscope (ESEM) equipped with an energy-dispersive X-ray spectroscopy (EDXS) probe. ESEM images were obtained using Everhart–Thornley (ETD) and circular backscatter (CBS) detectors for secondary electrons (SEs) and backscattered electrons (BSEs), respectively, at 10 kV and 20 kV. The voltages were chosen to obtain an optimal spatial resolution and EDXS analysis, respectively. The semi-quantitative EDXS analysis was performed by applying the Oxford Instruments EDS with Ultim Max SSD 40 mm^2^ detector and Oxford AZtec software (Oxford AZtecLive Version 6.1 March 2023, URL link: https://nano.oxinst.com/products/azteclive). Electron-backscatter diffraction (EBSD) analysis was performed using an SEM FSEM JEOL 7600F equipped with an EBSD detector. The obtained data were processed using the ICSD 2003 database for wulfenite as a reference.

Atomically resolved high-angle annular dark-field scanning transmission electron microscopy (HAADF-STEM) images were acquired via a probe Cs aberration correction transmission electron microscope (TEM) (JEM-ARM 200CF; JEOL, Tokyo, Japan). This microscope, also equipped with a JEOL Centurio 100 mm^2^ EDXS detector, operates at 200 keV and provides a spatial resolution of 0.08 nm in STEM mode. EDXS elemental maps were produced in STEM mode (STEM-EDXS) to evaluate the elemental distribution with a high spatial resolution.

The specimen preparation for the SEM/EDXS and TEM analyses involved cutting crystal samples along the principle crystallographic zone axes, which was determined using the EBSD technique. SEM/EDXS specimens were cut parallel to [001], set in technical epoxy resin pallets, polished, and coated with a 3 nm-thick C-coating.

For atomic-scale structural analysis, thin electron-transparent lamellae from both bipyramidal and tabular specimens of wulfenite were produced by using a focused ion beam scanning electron microscope (FIB-SEM; Helios NanoLab 600i dual beam system, Thermo Fisher Scientific, previously FEI Company, Eindhoven, the Netherlands). The TEM electron transparent lamellae were cut parallel to the (100) atomic plane.

## Results

### SCXRD

**Figure 2:**
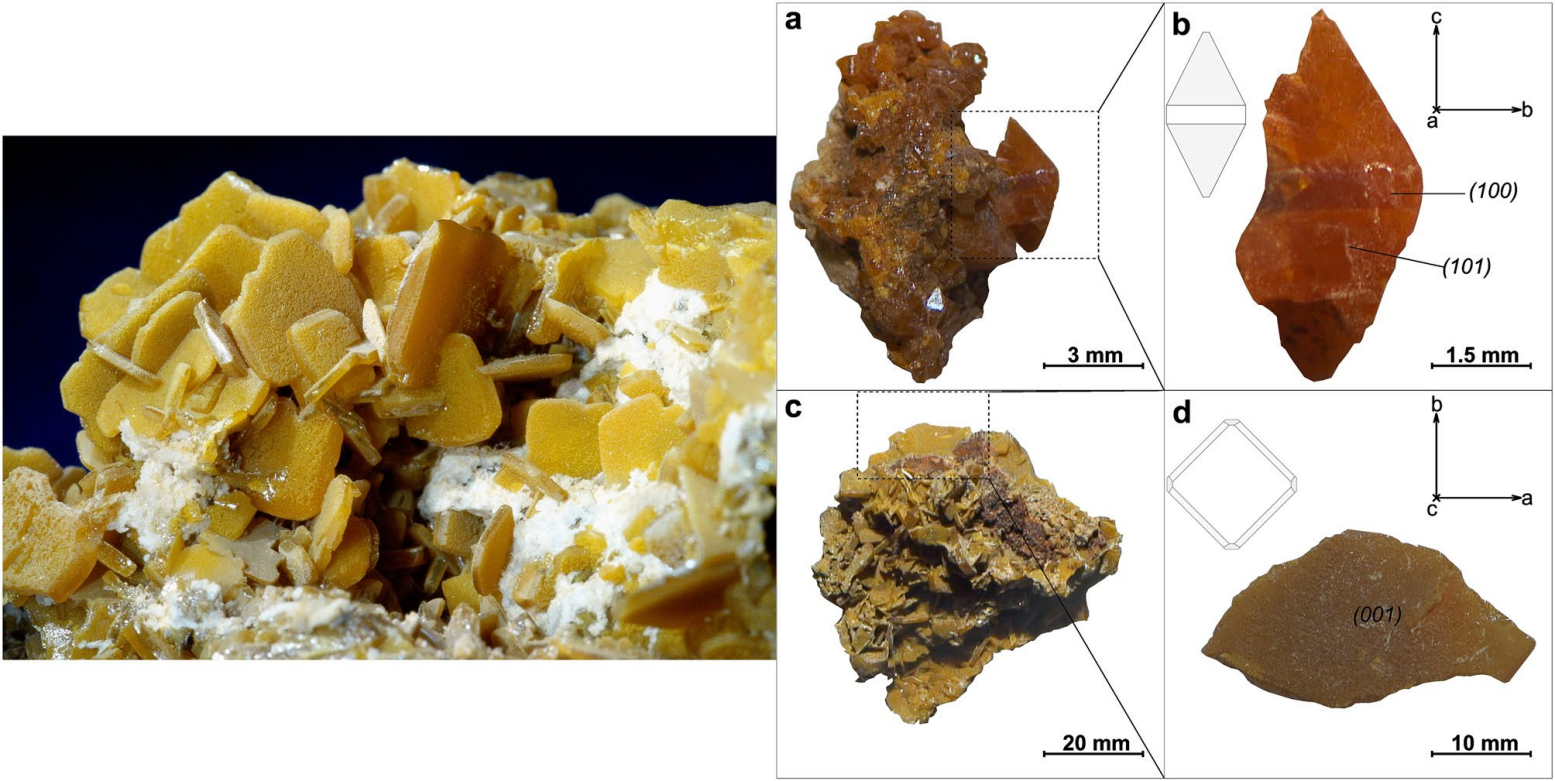
On the left is a photo of a heap of tabular wulfenites from Mežica. On the right are the clusters and specimens used in this study. (**a**) Cluster of orange translucent bipyramidal crystals, (**b**) bipyramidal specimen taken from the cluster, (**c**) cluster of yellowish translucent tabular wulfenite crystals, and (**d**) tabular specimen taken from the cluster shown in panel c.

The complete structural refinement data for the bipyramidal and tabular specimens are given in Tables 1 and 2, respectively. Both specimens belong to space group I4_1_/a which is the generally accepted space group for wulfenites^13^, in contrast to Cora et al.^9^, who reported a wulfenite specimen from Mežica to belong to the $\overline{4}{\text{I}}$ space group. No twinning was observed. The refinement data indicate a pure PbMoO_4_ composition, but other trace elements could be present below the detection limit. Atom sites and occupation factors for both specimens are given in Table 3. The occupation factors for Mo and Pb are very close to 1, which means that these positions are almost exclusively occupied by Mo and Pb atoms, and even if some substitutions may be present these are very rare. Crystal structure constructed from refinement data is shown in Fig. 2.

**Table 1:** SCXRD crystal data for bipyramidal specimen, along with data acquisition and structure refinement parameters.

Bipyramidal specimen crystal data	
Chemical formula	MoO_4_Pb
Formula weight	367.13 g/mol
Temperature	296 (2) K
Wavelength	0.71073 Å
Crystal size	0.020 × 0.020 × 0.030 mm
Crystal habit	Orange translucent pyramid
Crystal system	Tetragonal
Space group	I 4_1_/a (n°88)
Unit cell dimensions	a = 5.4317 (8) Å α = 90°
	b = 5.4317 (8) Å β = 90°
	c = 12.1144 (19) Å γ = 90°
Volume	357.42 (12) Å^3^
Z	4
Density (calculated)	6.823 g/cm^3^
Absorption coefficient	50.395 mm^−1^
F (000)	624
Data collection and structure refinement	
Theta range for data collection	4.11–55.55°
Index ranges	− 12 < = h < = 12, − 12 < = k < = 12, − 27 < = l < = 28
Reflections collected	15,252
Independent reflections	1169 [R (int) = 0.0495]
Coverage of independent reflections	99.7%
Absorption correction	Multi-scan
Max. and min. transmission	0.4320 and 0.3130
Refinement method	Full-matrix least-squares on F^2^
Refinement program	SHELXL-2014/7
Function minimized	Σ w (Fo^2^ -Fc^2^)^2^
Data/restrains / parameters	1169/0/15
Goodness-of-fit on F^2^	1.104
Final R indices	967 data; I > 2σ (I) R1 = 0.0161, wR2 = 0.0376
	All data R1 = 0.0217, wR2 = 0.0406
Weighting scheme	w = 1/[σ^2^(F_o_^2^) + (0.0174P)^2^ + 0.0993P]
	where P = (F_o_^2^ + 2F_c_^2^)/3
Extinction coefficient	0.0051 (3)
Largest diff. peak and hole	1.938 and − 2.795 eÅ^−3^
R.M.S. deviation from mean	0.301 eÅ^−3^

**Table 2:** SCXRD crystal data for tabular specimen, along with data acquisition and structure refinement parameters.

Tabular specimen crystal data	
Chemical formula	MoO_4_Pb
Formula weight	367.13 g/mol
Temperature	293(2) K
Wavelength	0.71073 Å
Crystal size	0.050 × 0.050 × 0.060 mm
Crystal habit	Orange translucent prism
Crystal system	Tetragonal
Space group	I 41/a
Unit cell dimensions	a = 5.4347(5) Å α = 90°
	b = 5.4347(5) Å β = 90°
	c = 12.1085(13) Å γ = 90°
Volume	357.65 (12) Å^3^
Z	4
Density (calculated)	6.818 g/cm^3^
Absorption coefficinet	50.364 mm^−1^
F (000)	624
Data collection and structure refinement	
Theta range for data collection	4.11–68.87°
Index ranges	− 13 < = h < = 14, − 13 < = k < = 14, − 30 < = l < = 31
Reflections collected	14,208
Independent reflections	1690 [R (int) = 0.0875]
Coverage of independent reflections	99.2%
Absorption correction	Multi-scan
Max. and min. transmission	0.1870 and 0.1520
Refinement method	Full-matrix least-squares on F^2^
Refinement program	SHELXL-2014/7
Function minimized	Σ w (Fo^2^ − Fc^2^)^2^
Data/restraints/parameters	1690/0/15
Goodness-of-fit on F^2^	1.046
Final R indices	1304 data; I > 2σ (I) R1 = 0.0327, wR2 = 0.0844
	All data R1 = 0.0431, wR2 = 0.0923
Weighting scheme	W = 1/ [σ^2^ (F_o_^2^) + (0.0305P)^2^]
	where P = (F_o_^2^ + 2F_c_^2^)/3
Extinction coefficient	0.0031 (6)
Largest diff. peak and hole	6.744 and − 3.035 eÅ^−3^
R.M.S. deviation from mean	0.635 eÅ^−3^

**Table 3:** Atom sites with respective coordinates and given occupation factors for each specimen.

Specimen	Atom	Site	x	y	z	Occupation factor
Bipyramidal	Mo	4a	0	1/4	1/8	0.991
	Pb	4b	1/2	3/4	1/8	1.005
	O	16f.	0.7640	0.3864	0.0441	1
Tabular	Mo	4a	0	1/4	1/8	1.003
	Pb	4b	1/2	3/4	1/8	1.000
	O	16f.	0.7639	0.3868	0.0439	1

### SEM/EDXS

**Figure 3:**
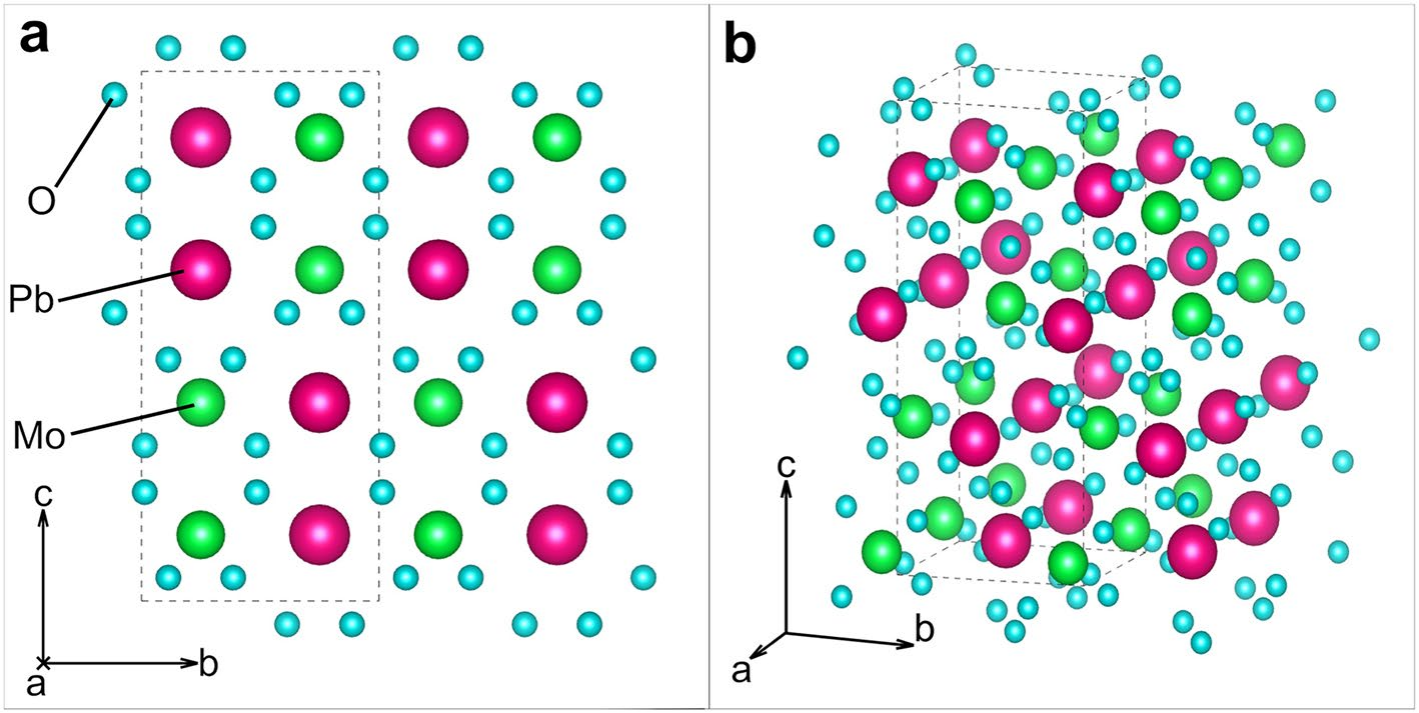
The crystal structure constructed from the refinement data. (**a**) View from [001] zone axis; (**b**) view from [111] zone.

The cross-sectional surfaces of the wulfenite crystals show the presence of numerous inclusions and pores, which are present in both bipyramidal and tabular specimens. These inclusions were identified by EDXS as calcite/aragonite (CaCO_3_), Pb-Fe oxides and rarely dolomite (CaMg(CO_3_)_2_)/Mg rich calcite, Pb oxides (PbO), and descloizite ((Pb,Zn)_2_VO_4_(OH)) (Table 4). The distribution of inclusions is not homogeneous; instead, they usually form layers or clusters, more commonly in the central part of the bipyramidal crystals, and they tend to form boundaries between the tabular base of the bipyramidal crystal and pyramidal outgrowths (Fig. 3). By far the most common inclusions are the calcite inclusions, which are a few tens of micrometres in size and range from subhedral to anhedral. Calcite inclusions, which form a distinct layer, are characterised by densely populated dark grey spots in BSE images (Fig. 3a) that are spread evenly along the {001} surfaces. Dolomite inclusions are less common, while other mineral inclusions are very sporadic. These bands are more common in the bipyramidal crystals and, when viewed in visible light, mark the edge of the much darker central portion of the crystal. Descloizite inclusions are rarely present (Fig. 3a); they are characterised by their small size and distinct texture. Descloizite inclusions rarely exceed 10 µm in diameter, and the crystals range from euhedral to anhedral. They usually occur together with calcite and dolomite in the abovementioned layers or clusters.

**Table 4:** Semi-quantitative chemical composition of bipyramidal wulfenite and its inclusions, given in wt%.

	Wulfenite	Calcite	Dolomite	Pb-Fe oxides	Pb-oxides	Descloizite
O	14.7	50.8	54.2	26.7	15.1	12.46
Mo	25.6			0,9		
Pb	59.8			18.4	84.9	57.81
Ca		49.2	29.2	0.65		
Mg			16.7			
Al				1.0		
Si				1.9		
V						12.0
Fe				44.8		0.8
Zn				4.3		16.0
As				0.7		0.9
Cr				0.41		
P				0.27		
Total	100.0	100.0	100.0	100.0	100.0	100.0

The data are from the EDXS analysis, and they are normalised.

### HAADF-STEM

**Figure 4:**
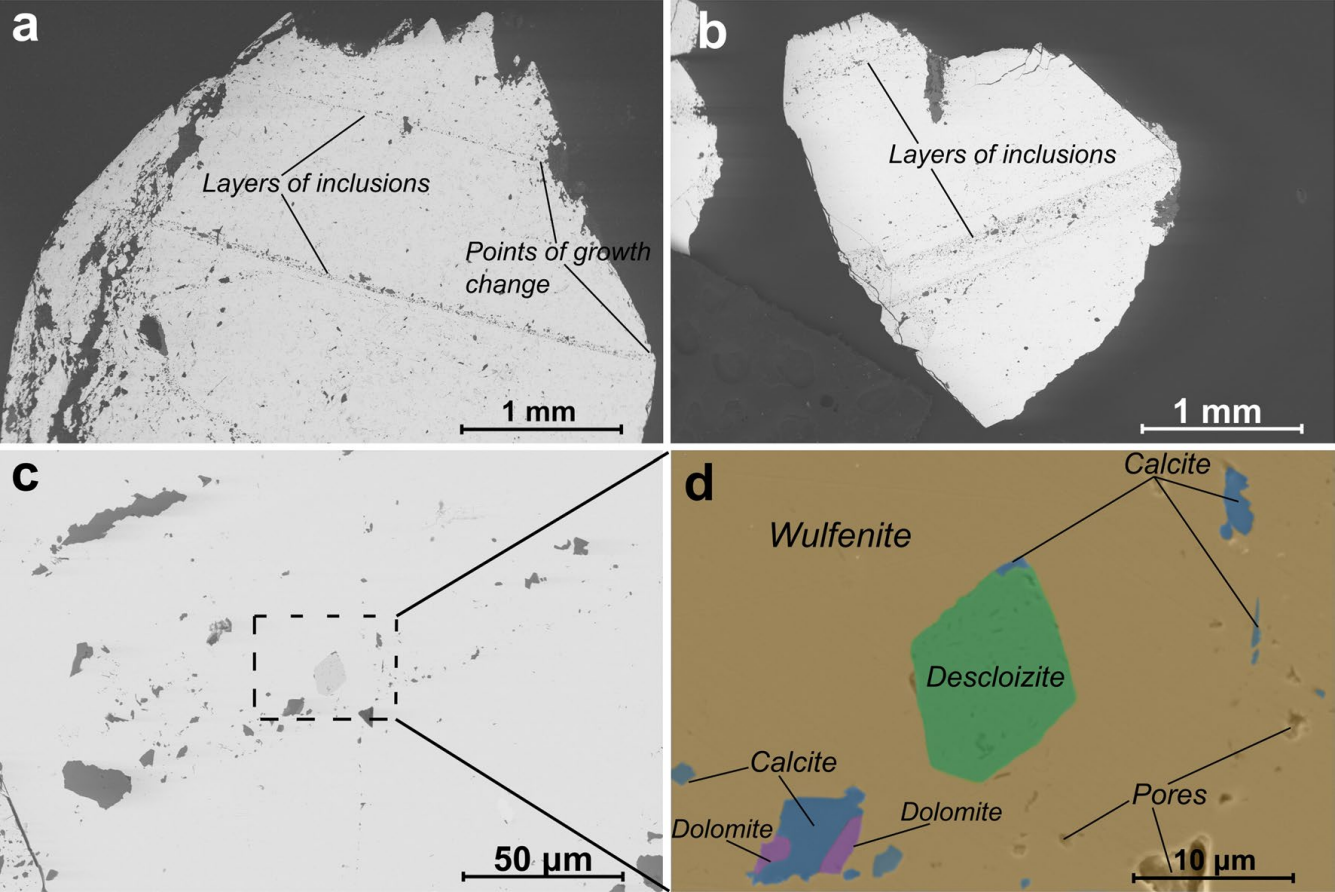
(**a**) and (**b**) Low-magnification SEM backscattered electron images showing layers of inclusions in the two bipyramidal specimens, where the growth change from tabular to bipyramidal can be observed on both images; the zone axis is [100]. (**c**) Higher magnification SEM BSE image showing a cluster of inclusions. (**d**) A closer look at those inclusions. The image is a colour-coded phase map created from EDXS analysis data, focusing on the rare descloizite inclusion. The orange colour represents the wulfenite phase, green represents descloizite, blue represents calcite, and magenta represents dolomite.

Figure 4a,b shows the bipyramidal and tabular crystals in their natural form. The crystal planes are marked on each crystal sample to indicate from where the TEM-FIB lamellae were cut. The inset shows the prepared FIB-cut lamella perpendicular to the a-axis directions. The STEM-EDXS analysis was performed on these lamellae to determine the chemical composition. The elements Mo, Pb, and O were detected, and their EDXS maps are shown in Fig. 4c. The ADF image shows the area where these maps were recorded. In the elemental maps, it can be seen that the elements are evenly distributed over the studied area. This shows that the studied lamella was obtained from a pure crystal without the impurities present in the samples. The slight variation in the intensity of the element maps in the lower right corner is due to the thickness variation, which is also observed on the ADF image. The atomic structure model of wulfenite projected along the a-axis is shown in Fig. 4d. The green, magenta, and blue spheres represent Mo, Pb, and O atoms, respectively. The dashed rectangle marks a unit cell. Points 4a, 4b, and 16f. mark the Wyckoff positions of the corresponding Mo, Pb, and O atoms. Figure 4e shows a representative HAADF-STEM high-magnification image (often referred to as a *Z*-contrast image) of the MoPbO_4_ crystal, as seen along the [100] zone axis. No obvious structural defects are seen in the observed sample regions. A close-up representative experimental image of the MoPbO_4_ crystal at high magnification and atomic resolution (HAADF-STEM) is shown as an inset in Fig. 4e. The contrast variation in the atomic resolution *Z*-contrast images roughly follows the *Z*^2^ dependence on the average atomic number of the studied atomic column, which makes the interpretation of both the atomic types and the atomic arrangements of the underlying crystal lattice relatively easy. In this respect, the highest contrast points in Fig. 4e are associated with the fully occupied Pb atomic columns (*Z* = 74), and the Mo atomic columns (*Z* = 42) are shown as spots of lower contrast. The Pb and Mo atomic columns are clearly visible in the experimental HAADF-STEM image, while the O atomic columns (*Z* = 8) are not visible due to their low atomic weight. The constructed atomic model for the [100] zone axis is superimposed on the atomically resolved HAADF-STEM image and agrees well with the underlying structure. The given value of 3.15 Å refers to the lattice spacing between the atomic planes along the c-axis.

## Discussion

**Figure 5:**
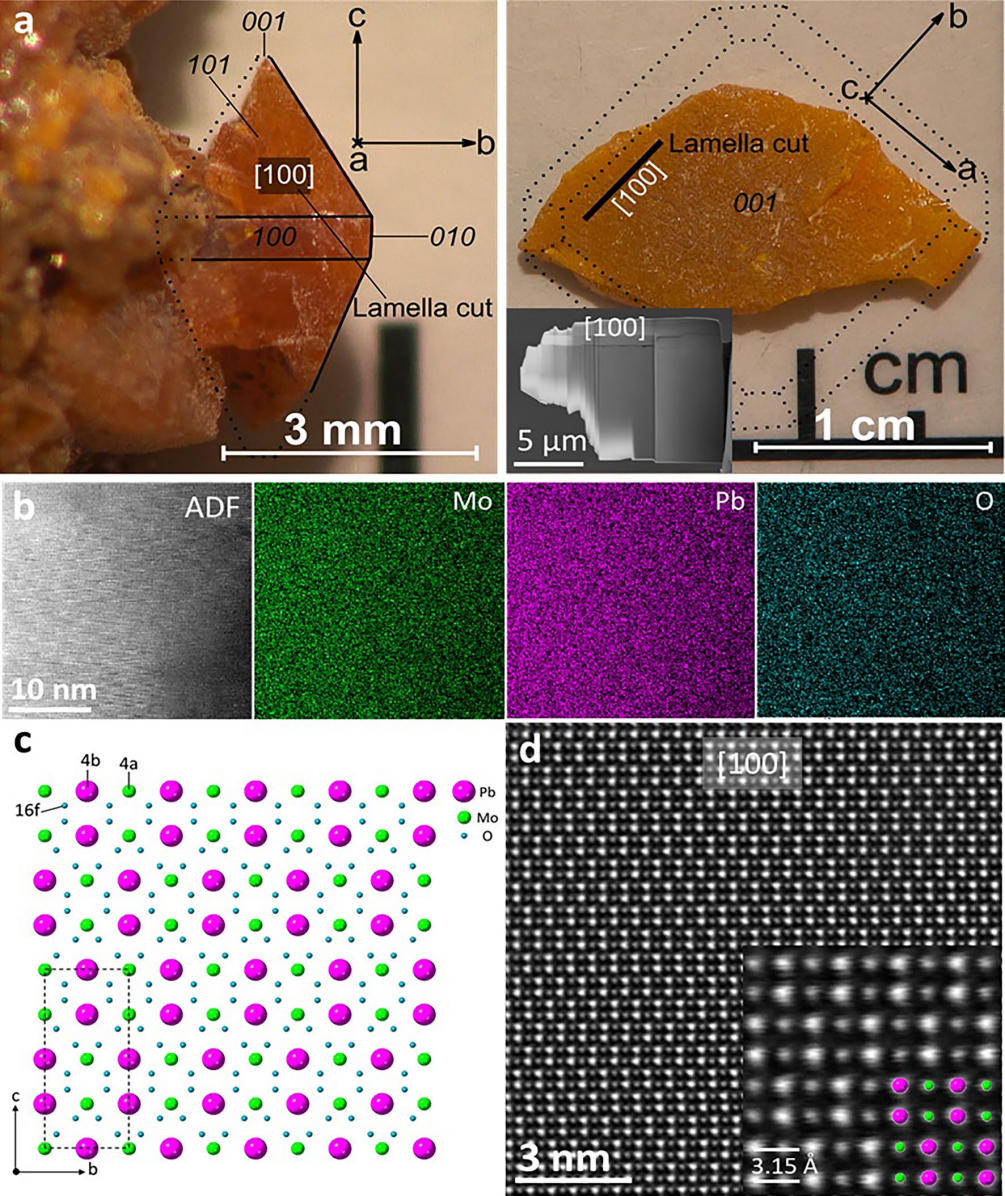
(**a**) Bipyramidal and tabular (**b**) crystals of the wulfenite sample. The lattice planes are marked on the crystals. The FIB-cut [100]-oriented lamella is shown in the inset obtained from tabular crystal. (**c**) STEM-EDXS elemental maps of Mo, Pb, and O obtained from the same area as the presented ADF image showing a homogeneous elemental distribution in the studied area. (d) Constructed atomic model of the MoPbO4 crystal (data from SCXRD analysis) projected along the a-axis. The green, magenta, and blue spheres represent Mo, Pb, and O atoms, respectively. The dashed rectangle marks a unit cell. Points 4a, 4b, and 16f mark the Wyckoff positions of the corresponding Mo, Pb, and O atoms. (**e**) Atomically resolved, experimental, unprocessed HAADF-STEM image at high magnification, projected along the a-axis ([100] zone axis). The high-resolution HAADF-STEM image with the superimposed atomic model viewed along the [100] zone axis is displayed in the inset. The lattice spacing between the atomic planes along the c-axis is shown (3.15 Å)

Wulfenite from the Mežica Mine is a secondary mineral precipitated from meteoric solutions during and after the oxidation of the primary mineral assemblage in the mine, which consisted mainly of galena (PbS) and sphalerite (ZnS). Galena minerals have inclusions of molybdenite (MoS_2_), while sphalerite minerals have Mo impurities of around 3 ppm^16,17^. During the growth of the wulfenite crystals, many inclusions, mostly of calcite and dolomite, were incorporated into the crystals themselves. These inclusions probably precipitated out of solution along with the wulfenite and, apparently, this occurred more frequently in the early stages of wulfenite crystal growth, right after the formation of the tabular base. This increased concentration of inclusions was predominantly found within a 200 µm thin layered segment or inclusion-rich layer within the wulfenite crystal. These layers or clusters of carbonate inclusions possibly indicate a change in the chemical composition of the solutions from which they were precipitated, as shown in Figs. 3 and 5. In addition, the presence of these inclusions likely affects the color and light transmission properties of the wulfenite crystals by altering their optical properties. In contrast to the work of Kloprogge et al.^18^, we have not found a secondary wulfenite phase that forms these darker layers. Instead, optically darker layers could be the result of visible light being scattered by the aforementioned inclusions. These layers and collections of inclusions in bipyramidal crystals mark the point where the {100} surfaces merge into the {101} surfaces and form the tetragonal pyramid on either side of the tabular base (see Fig. 3).

The diversity of crystal morphologies results either from internal factors such as the crystal structure and the presence of dislocations or twins or external factors such as the temperature, the pressure, the chemical composition of solutions, their degree of saturation, and Eh–pH conditions^7^.

Twinning or structure defects can be ruled out as a possible explanation for the differences in the external morphology of wulfenite, as they could not be detected by SCXRD or HAADF-STEM.

The structure of a crystal can be influenced by the presence of impurities resulting from the substitution of ions. This is because the different ionic radii and bond lengths associated with these impurities can alter the shape of the ionic polyhedra, leading to significant changes in the lattice parameters. Such changes can lead to a reduction in crystal symmetry and a transition to a space group with lower symmetry, such as ${\text{I}}\overline{4}$. At the same time, changes in the external shape of a crystal are often associated with a process called surface capping, in which the introduction of certain chemicals alters the surface energies of the crystal facets^19^. These changes in surface energies can promote the development of new crystal facets and cause the crystal to take on a different habit or crystal form. This process can significantly change the external appearance of the crystal. In our study, carbonates formed shortly after the initial development of the tabular wulfenite crystals. These carbonates are the only evidence of external influences in the solution, suggesting that they may have influenced the growth of the wulfenite crystals. However, we believe that this was not the case. Had surface capping occurred, we would expect the wulfenite crystals to show alternating growth patterns, alternating between tabular and bipyramidal shapes, such as in the spinel system (MgAl_2_O_4_) in the presence of BeO^20^. We would also expect to be able to detect these agents in the atomic resolution HAADF-STEM images, particularly outside of the thin inclusion-rich layer mentioned earlier. However, these images show no defects or impurities, supporting our conclusion that surface coverage did not significantly affect crystal growth. Moreover, the possible presence of impurities in our study does not seem to alter the crystal symmetries of the bipyramidal and tabular samples to the extent that they could be considered as different crystallographic phases, since both morphologies belong to space group I4_1_/a, as confirmed by SCXRD analysis.

**Figure 6:**
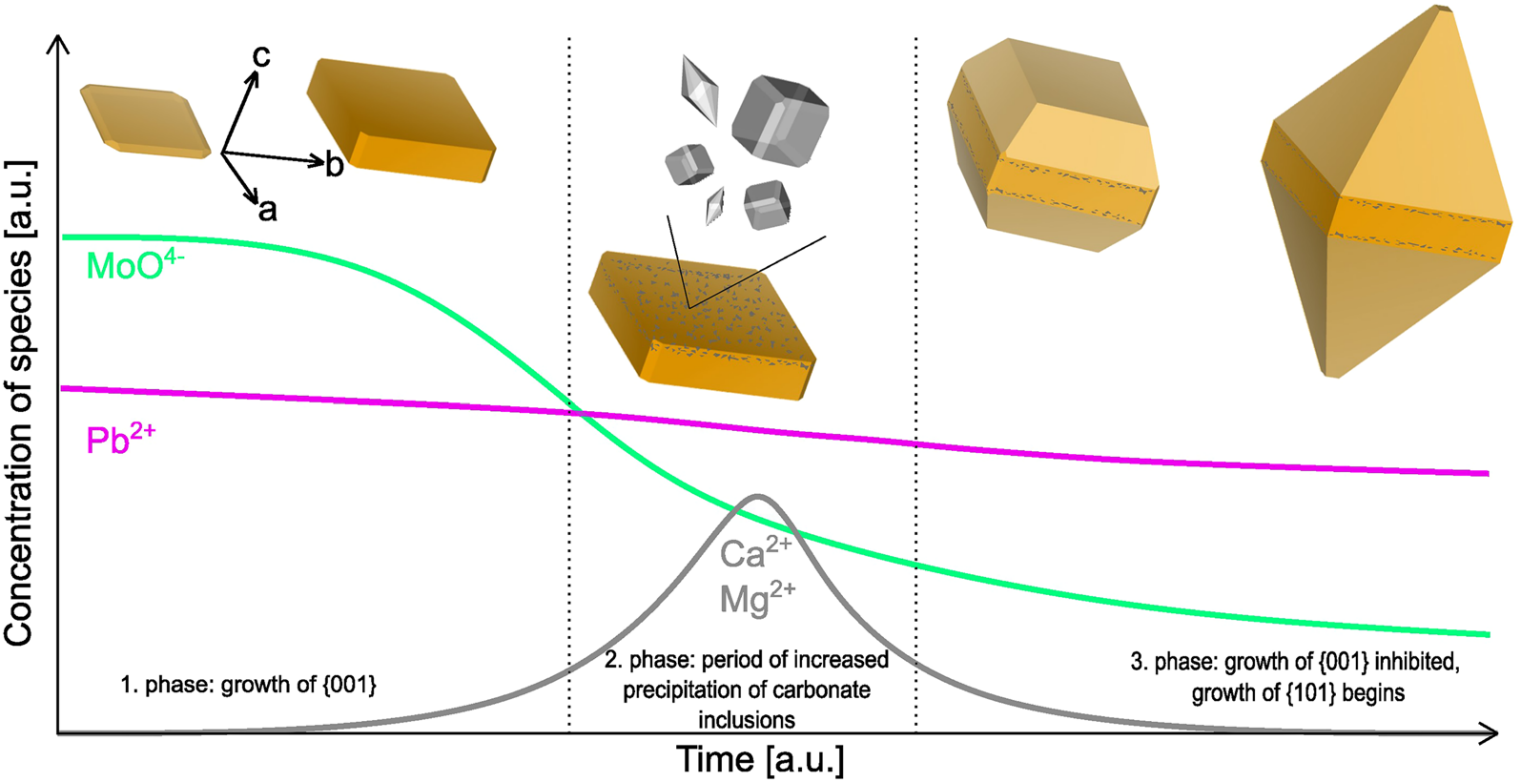
Concentrations of MoO_4_^2−^, Pb^2+^, and Ca^2+^ over time. The changes in concentrations mark the beginning and end of each growth phase of the wulfenite crystals.

While Vesselinov’s 1995 paper^13^ shows bipyramidal crystals as two tetragonal pyramids whose bases ((00–1) and (001)) are fused together and lack {100} facets, the bipyramidal specimens from Mežica usually exhibit {100} facets, along with {101} and often {001} at the very top and bottom of the crystals (Fig. 6). Bipyramidal specimens thus have a tabular base, with two tetragonal pyramids on each side ((001), (00–1)). Therefore, we can assume that the wulfenite crystals in Mežica started to grow as crystals with a tabular morphology, that means growth initially started primarily in directions < 100 > and was supressed in the directions < 001 > . However, at some point during crystal growth the main directions of growth changed to < 001 > and therefore some crystals began to develop {101} surfaces and eventually acquired a bipyramidal morphology. The others continued to grow as tabular crystals; in some of them, growth was suppressed, and they remained tabular. Vesselinov explains the change in preferred directions of growth with different relative concentrations of Pb^2+^ and MoO_4_^2−^ ions in the solutions from which wulfenite crystals precipitate^5,13^. With experimental work, Vesselinov showed that wulfenite crystals grow mainly in the directions < 100 > and form tabular crystals {001} when the concentration of MoO_4_^2−^ ions is greater than the concentration of Pb^2+^ ions in the solution (C_Pb_/C_MoO4_ < 1)^13^. If, on the other hand, the concentration of Pb^2+^ ions is higher (C_Pb_/C_MoO4_ > 1), wulfenite crystals preferably grow in directions < 001 > and form bipyramidal crystals in which two tetragonal pyramids {101} are fused in the 001 plane^13^.

Consequently, we propose a mechanism in which the relatively rapid change in the chemical composition of the solutions during the growth of the wulfenite crystals is the most important factor determining the morphology of the wulfenite crystals from Mežica, with the abovementioned work of Vesselinov providing the theoretical basis. The crystallisation of wulfenite can be represented with three distinct phases of crystal development (see Fig. 5):

- 1. During the first phase (Fig. 4: Growth of {001} crystals) of wulfenite formation in the Mežica area, the solutions were rich in Pb^2+^ and MoO_4_^2−^ ions from the oxidation of galena (as the main source of Pb)^16^, sphalerite and molybdenite (as the main source of Mo)^16,17^. The solutions penetrated systems of faults and fractures in the carbonate host rock and rapidly became supersaturated, resulting in the precipitation of wulfenite. The specimens studied in this work all began to grow primarily in < 100 > directions and formed small, tabular {001} crystals, indicating a ratio C_Pb_/C_MoO4_ < 1^13,21^. Inclusions also grew in these crystals, mainly consisting of calcite, suggesting the presence of Ca and Mg ions in solution. In addition, the presence of carbonates also allows conclusions to be made about the pH of the solutions, which was in the range of 7.5–8, as predicted for sulphate-hydrocarbonate solutions at ambient temperatures (< 50 °C)^11^.
- 2. In the second, relatively short phase (Fig. 4: The period of the increased precipitation of carbonate inclusions), heterogeneous growth of carbonate inclusions increases rapidly. This abrupt increase in carbonate precipitation marks the change in the chemical composition of the solutions, where the ratio C_Pb_/C_MoO4_ ratio becomes greater than 1^13,21^. It should be noted that we assume that the carbonate precipitation coincides with the change in the composition of the solutions. It is therefore the consequence of the change in solution chemistry and not its cause. Consequently, the carbonate precipitation has no influence on the further development of the wulfenite crystals. The shift in the C_Pb_/C_MoO4_ ratio is probably due to the reduced concentration of MoO_4_^2−^ in the solutions, which is due to extensive oxidation of the sphalerite ore bodies, depleting the primary source of Mo^17^. In contrast, the concentration of Pb^2+^ remained stable as long as there was sufficient interaction between galena and meteoric water, as galena ore bodies oxidised less rapidly than sphalerite ore bodies^17^. The carbonate inclusions formed thin bands that covered the {001} surfaces of the wulfenite crystals, with fewer inclusions in the intermediate areas. This indicates that the elevated precipitation rates of Ca and Mg were not uniform, probably due to fluctuating concentrations of dissolved Ca and Mg over a relatively short geologic period. However, the rate of precipitation of carbonate inclusions in this phase was consistently higher than in earlier and later phases, as shown in Fig. 3.
- 3. In the third phase, the ratio of C_Pb_/C_MoO4_ in the solutions is greater than 1, which promotes the growth of wulfenite, especially in the directions < 001 > , so that tetragonal pyramids {101} began to grow on the tabular base of the crystals, giving them bipyramidal morphology (Fig. 4: Growth of {001} inhibited, growth of {101} begins).

Some crystals retain their tabular morphology during the third phase. It should be noted that carbonate inclusions are also present in these crystals in the form of bands, suggesting that they precipitated from the same solutions as bipyramidal wulfenite but did not experience surface growth due to factors such as orientation or spatial limitations that did not allow growth in the < 001 > directions. This explains why some clusters show both morphologies, although the crystals are spatially very close to each other, separated by distances of at most a few millimetres.

## Conclusions

Wulfenite crystals from the Mežica Mine exhibit bipyramidal and tabular morphologies. The bipyramidal crystals consist of a tabular base and two tetragonal pyramids that grow on opposite sides of the base. The following conclusions can be drawn:

- The bipyramidal and tabular crystals are chemically pure wulfenite (PbMoO_4_), as confirmed by SEM-EDXS; however, each form contains several inclusions, mostly consisting of carbonates, Pb-Fe oxides, Pb oxides and rarely descloizite. These inclusions are often arranged in layers.
- These layers occur throughout the tabular crystals and in the tabular base of the bipyramidal crystals.
- The SCXRD analysis confirmed that both forms belong to the same space group, I4_1_/a, with no twinning. The analysis also confirmed the pure wulfenite compositions determined from the EDXS, which were further confirmed by the HAADF-STEM analysis.
- The differences in morphology can therefore be attributed to changes in composition (concentrations of Pb^2+^ and MoO_4_^2−^) during precipitation from the meteoric solution rather than to differences in lattice geometry, defects such as twinning or surface capping by carbonates.
- We propose a growth mechanism that consists of three phases of growth:

1. During the first phase, the concentration ratio between C_Pb_ and C_MoO4_ in the solution is lower than 1, which favours the growth of tabular crystals.
2. In the short second phase, carbonates precipitated from the solutions and are present as inclusions in the wulfenite. Carbonate precipitation occurs incidental with the change in the concentrations of Pb^2+^ and MoO_4_^2−^ (C_Pb_/C_MoO4_ ratio becomes greater than 1).
3. In the third phase the C_Pb_/C_MoO4_ > 1 ratio favours the pyramidal growth of wulfenite crystals. As some of the tabular crystals remained protected from the new influx of solutions, they maintained their tabular morphology, while others acquired a bipyramidal form.

## Data Availability

The data are available in the Department of Geology, Faculty of Natural Sciences and Engineering, University of Ljubljana and in the Department for Nanostructured Materials, Jozef Stefan Institute (contact person Nastja Rogan Šmuc, nastja.rogan@ntf.uni-lj.si).
