# Publisher Correction: Formation mechanisms and environmental influences on the crystal growth of wulfenite

**DOI:** 10.1038/s41598-024-60770-8

**Published:** 2024-05-02

**Authors:** Nik Gračanin, Matejka Podlogar, Sorour Semsari Parapari, Pascal Boulet, Francisco Ruiz-Zepeda, Sašo Šturm, Nastja Rogan Šmuc

**Affiliations:** 1 Department for Nanostructured Materials, Jozef Stefan Institute, Jamova Cesta 39, 1000 Ljubljana, Slovenia; 2 Department of Geology, Faculty of Natural Sciences and Engineering, University of Ljubljana, Aškerčeva 12, 1000 Ljubljana, Slovenia; 3 Institut Jean Lamour (UMR CNRS 7198), Centre de Compétences XGamma, 2, Allée André Guinier, 54011 Nancy, France; 4 Department of Materials Chemistry, National Institute of Chemistry, Hajdrihova 19, 1000 Ljubljana, Slovenia

Correction to: *Scientific Reports* 10.1038/s41598-024-60043-4, published online 22 April 2024

The original version of this Article contained errors in the Figure legends of Figures 1, 2, 3, 4, 5 and 6. The legends of these Figures were inadvertently switched.

As a result, the legend of Figure 1,

“On the left is a photo of a heap of tabular wulfenites from Mežica. On the right are the clusters and specimens used in this study. (**a**) Cluster of orange translucent bipyramidal crystals, (**b**) bipyramidal specimen taken from the cluster, (**c**) cluster of yellowish translucent tabular wulfenite crystals, and (**d**) tabular specimen taken from the cluster shown in panel c.”

now reads:

“Schematic representation of the two most common morphological varieties of wulfenite crystals, (**a**) bipyramidal and (**b**) tabular crystals, with indexed surfaces. Ideal wulfenite crystal structure viewed along the (**c**) [100] and (d) [001] zone axes, respectively.”

The legend of Figure 2,

“The crystal structure constructed from the refinement data. (**a**) View from [001] zone axis; (**b**) view from [111] zone axis.”

now reads:

“On the left is a photo of a heap of tabular wulfenites from Mežica. On the right are the clusters and specimens used in this study. (**a**) Cluster of orange translucent bipyramidal crystals, (**b**) bipyramidal specimen taken from the cluster, (**c**) cluster of yellowish translucent tabular wulfenite crystals, and (**d**) tabular specimen taken from the cluster shown in panel c.”

The legend of Figure 3,

“(**a**) and (**b**) Low-magnification SEM backscattered electron images showing layers of inclusions in the two bipyramidal specimens, where the growth change from tabular to bipyramidal can be observed on both images; the zone axis is [100]. (**c**) Higher magnification SEM BSE image showing a cluster of inclusions. (**d**) A closer look at those inclusions. The image is a colour-coded phase map created from EDXS analysis data, focusing on the rare descloizite inclusion. The orange colour represents the wulfenite phase, green represents descloizite, blue represents calcite, and magenta represents dolomite.”

now reads:

“The crystal structure constructed from the refinement data. (**a**) View from [001] zone axis; (**b**) view from [111] zone axis.”

The legend of Figure 4,

“(**a**) Bipyramidal and tabular (**b**) crystals of the wulfenite sample. The lattice planes are marked on the crystals. The FIB-cut [100]-oriented lamella is shown in the inset obtained from tabular crystal. (**c**) STEM-EDXS elemental maps of Mo, Pb, and O obtained from the same area as the presented ADF image showing a homogeneous elemental distribution in the studied area. (**d**) Constructed atomic model of the MoPbO_4_ crystal (data from SCXRD analysis) projected along the a-axis. The green, magenta, and blue spheres represent Mo, Pb, and O atoms, respectively. The dashed rectangle marks a unit cell. Points 4a, 4b, and 16f. mark the Wyckoff positions of the corresponding Mo, Pb, and O atoms. (**e**) Atomically resolved, experimental, unprocessed HAADF-STEM image at high magnification, projected along the a-axis ([100] zone axis). The high-resolution HAADF-STEM image with the superimposed atomic model viewed along the [100] zone axis is displayed in the inset. The lattice spacing between the atomic planes along the c-axis is shown (3.15 Å).”

now reads:

“(**a**) and (**b**) Low-magnification SEM backscattered electron images showing layers of inclusions in the two bipyramidal specimens, where the growth change from tabular to bipyramidal can be observed on both images; the zone axis is [100]. (**c**) Higher magnification SEM BSE image showing a cluster of inclusions. (**d**) A closer look at those inclusions. The image is a colour-coded phase map created from EDXS analysis data, focusing on the rare descloizite inclusion. The orange colour represents the wulfenite phase, green represents descloizite, blue represents calcite, and magenta represents dolomite.”

The legend of Figure 5,

“Concentrations of MoO_4_^2−^, Pb^2+^, and Ca^2+^ over time. The changes in concentrations mark the beginning and end of each growth phase of the wulfenite crystals.”

now reads:

“(**a**) Bipyramidal and tabular (**b**) crystals of the wulfenite sample. The lattice planes are marked on the crystals. The FIB-cut [100]-oriented lamella is shown in the inset obtained from tabular crystal. (**c**) STEM-EDXS elemental maps of Mo, Pb, and O obtained from the same area as the presented ADF image showing a homogeneous elemental distribution in the studied area. (**d**) Constructed atomic model of the MoPbO_4_ crystal (data from SCXRD analysis) projected along the a-axis. The green, magenta, and blue spheres represent Mo, Pb, and O atoms, respectively. The dashed rectangle marks a unit cell. Points 4a, 4b, and 16f mark the Wyckoff positions of the corresponding Mo, Pb, and O atoms. (**e**) Atomically resolved, experimental, unprocessed HAADF-STEM image at high magnification, projected along the a-axis ([100] zone axis). The high-resolution HAADF-STEM image with the superimposed atomic model viewed along the [100] zone axis is displayed in the inset. The lattice spacing between the atomic planes along the c-axis is shown (3.15 Å).”

The legend of Figure 6,

“Schematic representation of the two most common morphological varieties of wulfenite crystals, (**a**) bipyramidal and (**b**) tabular crystals, with indexed surfaces. Ideal wulfenite crystal structure viewed along the (**c**) [100] and (**d**) [001] zone axes, respectively.”

now reads:

“Concentrations of MoO_4_^2-^, Pb^2+^, and Ca^2+^ over time. The changes in concentrations mark the beginning and end of each growth phase of the wulfenite crystals.”

The original Article has been corrected.

